# An ultrasound limited test initiating medical airborne transportation (ULTIMAT-protocol): its impact in other settings in medicine

**DOI:** 10.1186/s13613-019-0620-x

**Published:** 2020-02-03

**Authors:** Daniel A. Lichtenstein

**Affiliations:** Medical Intensive Care Unit, Hospital Ambroise Paré (AP-HP), Paris-West university, 9 avenue Charles de Gaulle, F-92100 Boulogne (Paris-West), France

Critical medicine is sometimes performed in airborne missions. How far can ultrasound help in these settings? This Editorial is devoted to approach the most difficult, but the same use can be done in less extreme conditions, up to routine assessments in numerous disciplines.

The principle of an airborne medical evacuation is to improve the outcome for the patient by providing faster care. Such a procedure is challenging [1]. Restricted space, limited equipment, engine noise among other factors are hindrances for diagnosing an acute circulatory failure, a pneumothorax, etc. Even in a quiet hospital, doctors still request imaging to confirm their clinical diagnoses, sometimes initiating another kind of risky travel [2].

We have been using ultrasound in our airborne missions since 1995, as a natural complement to the physical examination in areas where it showed limited performances [3]. We use light devices (the 3500-g TM-18, Dymax, Pittsburgh, USA, 1994) (the 1850-g Tringa S-50, Pie Medical, Maastricht, Netherland, 1998) (the 180-g Signos RT, Signostics, Thebarton, Australia, 2011) (the 750-g U-lite, Sonoscanner, Paris, France, 2016) all equipped with microconvex probes. Other machines are available (Lumify, Butterfly, iViz, etc.), but we had no opportunity to test them (note that using these tools with traditional probes would be possible, but would not be stricto sensu our protocol). Our ultrasound limited test initiating medical airborne transportation is a whole-body approach focused on ten critical, selected targets using a single probe and without Doppler. In no step of our protocol, by definition, is Doppler required (users having Doppler are free to use it of course—once again, it would be a distinct protocol). Our protocol involves searching for floating DVT in strategic areas (Fig. 1 ). Simple emergency cardiac sonography includes pericardial state, right ventricle volume, rough real-time approach to left ventricular function; lung ultrasound helps by estimating pulmonary artery occlusion pressure based on the A-profile or B-profile [4] (Fig. 2). This help may be of interest, because sophisticated methods, including Doppler-echocardiography, are difficult to access here [5]. One checks for the absence of pneumothorax and few other items (Table. 1). In trained hands, a negative protocol is achieved in 1 min. Even multiplied by three or four for less trained users, the timing remains fully acceptable.

**Fig. 1:**
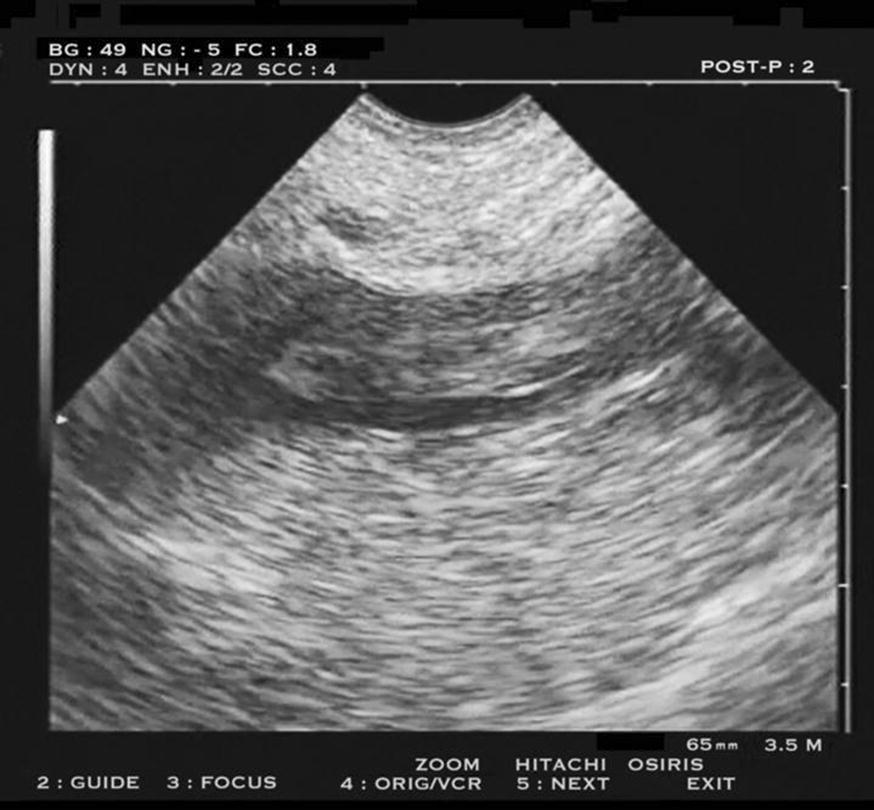
Deep venous thrombosis at the external iliac vein (long axis), perfectly visible using a microconvex probe. The pattern of floating thrombosis, typical here, is even more obvious on video. The volume is substantial enough for likely generating serious hemodynamic troubles in case of dislodgement, so this pattern should make reconsider the safety of the transportation

**Fig. 2:**
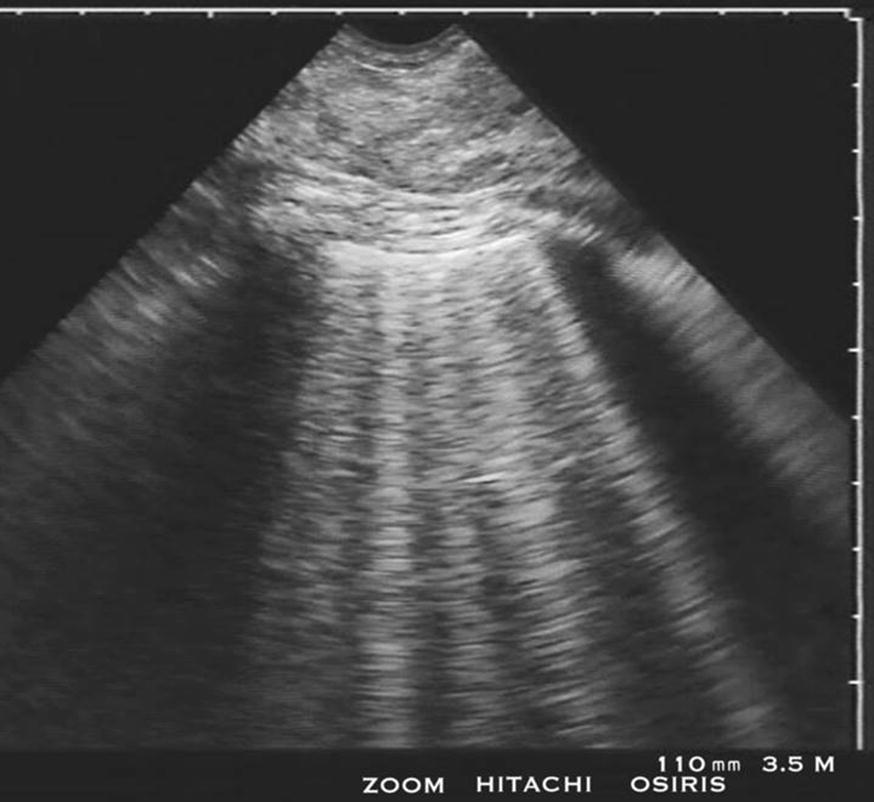
One main impact of the BLUE-protocol is the diagnosis of pulmonary edema, highly suggested when lung rockets are harmoniously distributed anteriorly. The association with lung sliding generates the B-profile, suggesting hemodynamic pulmonary edema with a 97% sensitivity and a 95% specificity

**Table 1:** The ten targets of our ultrasound protocol which follow the physical examination of a patient before an emergency airborne evacuation

Ultrasound targets	Disease	Notes
1. Anterior lung sliding	Search for pneumothorax	Rules out pneumothorax in a few seconds
2. Anterior lung rockets	Suggests hemodynamic pulmonary edema (patent or occult)	Rules out pneumothorax in a few seconds
3. Main veins patency	Deep venous thrombosis	Decreasing the risk of dislodgement, a likely event during turbulent transportation. A simple protocol allows fast screening on strategic areas
4. Pericardium	Pre-tamponade	Takes a few seconds
5. Right ventricle rough volume	May accompany pulmonary embolism	Can be done without Doppler
6. Left ventricle rough contractility	Left heart function	A reasonable assessment, between the limited physical examination and the comprehensive LV approach using Doppler. Lung ultrasound reinforces this basic approach
7. Bladder repletion	Urinary obstacle	Diagnosing a urinary obstacle before the transportation seems a fast, simple and basic step
8. Fluid in pleura	Many causes	A few seconds are required
9. Fluid in peritoneum	Incipient hemorrhage	A few seconds are required
10. Pneumoperitoneum	Incipient severe GI tract disorder	A few seconds are required

In scheduled missions without any apparent problem, the patient’s safety should be reconsidered if pathological conditions are present, particularly floating DVT. Clinical and biological items (kalemia, etc.) being considered apart, if all examinations are clear, the patient is decreed “sonographically fit-to-fly”. If unexpected dramas occur, the physician is armed for facing sudden respiratory failure using lung and venous ultrasound (BLUE-protocol), circulatory failure using a simple emergency cardiac sonography then lung ultrasound (FALLS-protocol) or cardiac arrest using a standardized approach beginning by lung ultrasound (SESAME-protocol) [4, 6, 7].

We use the principles of holistic ultrasound, favoring simple machines with minimal options, but including a “new” target, the lung. Lung and heart are the two mainstems of critical ultrasound [6, 8]. Both interact, lung ultrasound helps when cardiac windows are suboptimal. Our protocol fits perfectly into the spirit of holistic ultrasound: the adjunct of lung ultrasound, the development of an adapted venous ultrasound, the notion of ultrasound lung–heart synergy, all these concepts allow the use of simplicity. Our single and universal probe fits for the whole body. It is optimal for most critical targets (lungs, veins, abdomen) and is suitable for other critical areas, particularly the simple emergency cardiac sonography (Additional file 1: Video file 1, Additional file 2: Video file 2 and Additional file 3: Video file 3 ). This subtlety enables on-site use in extreme emergencies up to cardiac arrest [7]. This ability to use a small and light machine is an absolute necessity in this setting, among other life-support equipments, and above all, proves sufficient. Our current 750-g machine (probe included) can be stored in a 1820-cc box (Fig. 3).

**Fig. 3:**
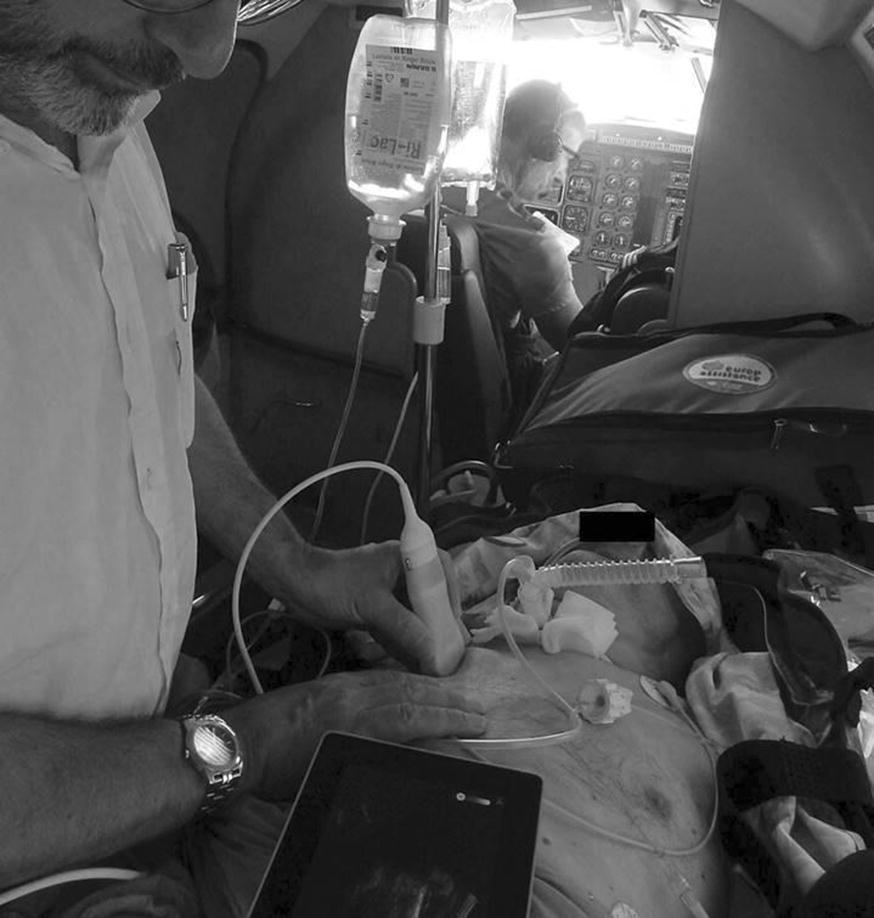
One single machine, well operated, can replace more than just radiography, even CT in strategic areas (lungs mainly). The small size, the light weight are mandatory criteria for life-saving use in this airplane, but the same spirit can be found at ground level, in ICUs or many other settings

Yet most of us are not working up at 30.000 feet. Let us consider other areas. The same visual approach can be done in any critically ill patient of any ICU. Simple machines and a simple technique can be used in the first minutes in most critical situations. Any unstable patient before a necessary transportation (e.g., to CT) may benefit from (part of) this fast protocol. Obviously, the user just needs to recognize, when the problem remains unsolved, the time to use more sophisticated approaches. Lung ultrasound, a major part of our protocol, is useful in numerous settings. We have listed 17 disciplines for potential applications, from medicalized ambulances, emergency rooms, pediatrics, cardiology, pulmonology, obstetrics, etc., up to family medicine [9]. Before the spectacular revolution of CT, lung ultrasound was operational for bedside use, with its familiar overwhelming advantages (cost, irradiation, point-of-care use). The possibility of using the same approach without any adaptation from the critically ill in airborne missions to the ambulatory patient, that is, locating these opposed settings on the same level of simplicity, is a perfect illustration of holistic ultrasound. This is maybe the most important message of this Editorial.

Mainly, this space was the opportunity for describing technical considerations (simple machine, universal probe) proving contributive in all concerned settings. Regarding airborne missions, we can hardly accept to be with a difficult patient 30.000 feet above the ocean and deprived of this visual assistance [10]. More than a tool, this use of ultrasound should be the physician’s best friend in any critical or routine work.

## Supplementary information

**Additional file** **1****: Video file 1.** Basic pattern of pneumothorax using our universal microconvex probe (step 1 of the SESAME-protocol, in cardiac arrest).

**Additional file** **2****: Video file 2.** Floating deep venous thrombosis (DVT) using our universal microconvex probe (step 2 of the SESAME-protocol, in cardiac arrest). An A-profile with a DVT is a basic profile of the BLUE-protocol, 99% specific to pulmonary embolism.

**Additional file** **3****: Video file 3.** A cardiac window using our universal microconvex probe (step 5 of the SESAME-protocol in cardiac arrest). Step 3 was a fast search of free blood (hypovolemic arrest). Step 4 was a fast search for pericardial tamponade. Just note that this probe allows to roughly assess the right ventricle volume, the left ventricle contractility (and the pericardial status).

## Data Availability

The datasets supporting the conclusions of this article are included within the article and its additional file.
